# Molecular spectrum of excision repair cross-complementation group 8 gene defects in Chinese patients with Cockayne syndrome type A

**DOI:** 10.1038/s41598-017-14034-3

**Published:** 2017-10-20

**Authors:** Xiaozhu Wang, Yu Huang, Ming Yan, Jiuwei Li, Changhong Ding, Hong Jin, Fang Fang, Yanling Yang, Baiyan Wu, Dafang Chen

**Affiliations:** 1 Department of Epidemiology and Biostatistics, School of Public Health, Peking University Health Science Center, Beijing, 100191 China; 2 Department of Medical genetics, School of Basic Medical Sciences, Peking University Health Science Center, Beijing, 100191 China; 3 Department of Neurology, Beijing Children’s Hospital, Capital Medical University, Beijing, 100045 China; 4 Departments of Paediatrics, Peking University First Hospital, Beijing, 100034 China

## Abstract

There are two genetics complementary groups Cockayne syndrome type A and B (CS-A and CS-B OMIM 216400, 133540), which is a rare autosomal recessive segmental progeroid syndrome. Homozygous or compound heterozygous mutations in the excision repair cross-complementation group 8 gene (*ERCC8*) result in CS-A, and mutations in *ERCC6* result in CS-B. Homozygous *ERCC6/ERCC8* mutations also result in UV-sensitive syndrome. In this study, twenty-one Han Chinese patients with CS were investigated to identify mutations in *ERCC8*/*ERCC6*, of which thirteen cases with CS-A were identified with the mutations of *ERCC8*. There are five types mutations of *ERCC8* in our study, such as exon 4 rearrangement, c.394_398delTTACA, c.299insA, c.843 + 2 T > C, and c.2 T > A. An estimated frequency of exon 4 rearrangement accounts for 69.23% and c.394_398delTTACA accounts for 11.53% in our cohort. Haplotype analysis revealed that the exon 4 rearrangement and c.394_398delTTACA mutations originated from a common founder in the Chinese population respectively. With the identification of three novel *ERCC8* mutations, this study expanded the molecular spectrum of known *ERCC8* defects, and furthermore, suggests that the exon 4 rearrangement and c.394_398delTTACA mutations may be a common underlying cause of CS-A in the Chinese population, which is different from that in other populations.

## Introduction

Cockayne Syndrome (CS OMIM 133540, 216400) is a rare segmental premature aging syndrome characterized by growth failure, lipodystrophy, mental retardation, brain dysmyelination with calcium deposits, sensorineural hearing loss, and cutaneous photosensitivity^1,2^. There are two genetically complementary groups of CS, type A (CS-A, OMIM 216400) and type B (CS-B, OMIM 133540), which show diverse clinical symptoms^3^. CS-A is caused by mutations in excision repair cross-complementation group 8 (*ERCC8*) and accounts for approximately 20% of characterized cases with CS^2^, however; a more recent study reported that CS-A accounted for approximately one third of CS cases^4^.

*ERCC8* (also known as *CSA*) is located on chromosome 5q12.1 and encodes a 44-kDa protein that contains 396 amino acids. CSA belongs to the WD40-repeat family of proteins which are highly conserved repeated units usually ending with Trp-Asp (WD). WD40-repeat proteins are found in all eukaryotes regulating a variety of cellular functions, such as cell-fate determination, gene transcription, mRNA modification, transmembrane signalling, and cell division^5–7^. ERCC8 contains five WD repeats based on amino-acid sequence, and another two WD-40 repeats have been predicted by sequence alignment with a structural template^5,8^. Structural analysis revealed that WD-40 domain exhibited a β-propeller architecture which usually comprised seven repeats, moreover CSA also comprised helix-loop-helix motif and seven WD40 propellers which were well ordered^9,10^.

Previous study indicated that CSA/ERCC8 and CSB/ERCC6 played differential roles in mammalian Transcription coupled nucleotide excision repair (TC-NER). When elongation RNA polymerase II (RNAPII0) was blocked and stalled in DNA lesion site, CSB was recruited to the lesion site, which attracted NER proteins, chromatin remodellers and the CSA-DDB1 E3-ubiquitin ligase complex as a repair coupling factor. CSA cooperated with CSB recruit XAB2, the nucleosomal binding protein HMGN1 and transcript elongation factor A (TFIIS)^11^. TC-NER is a complex procedure which requires two essential assembly factors (CSA and CSB), the core NER factors and TC-NER specific factors to repair transcription-blocking lesions without displacement of the DNA damage-stalled RNAPIIo^11,12^. CSA was integrated into a complex which contained cullin4A and Roc1 and displayed ubiquitin ligases via interaction with DDB1^10,13^. CSA was also found to be a subunit of an E3 ubiquitin ligase complex and CSB was a substrate of this E3 ubiquitin ligase^14^.

CS-A is a rare inherited disease with about 40 types of mutations in *ERCC8* reported worldwide (http://www.hgmd.cf.ac.uk/ac/gene.php?gene=ERCC8). Most cases of CS have different mutations in different populations, and some mutations found in *ERCC8* are unique in each population, and therefore, may have originated from a common founder mutation. In fact, some founder mutations may be the major cause of this disorder in certain populations. For example, the p.Tyr322X mutation found among Christian Arabs in Northern Israel has a high carrier frequency (about 6.79%) that was demonstrated to originate from a common founder^15^. In an East-Asian population, Ren *et al*. found three novel mutations and identified a rearrangement mutation in exon 4 as a major cause of CS-A in five patients from Japan^16^. In this study, the authors proposed that the exon 4 rearrangement may have come from a common founder mutation, but they did not investigate further.

Prior to this study, there has been no systematic investigation of *ERCC8* in a Chinese Han population. A number of Northern Chinese cases of CS-A have been collected in several hospitals in Beijing which enabled for us to perform a genetic analysis. Therefore, in the present study, we specifically investigated the spectrum of *ERCC8* mutations associated with CS-A in a Northern Chinese population, which is the first such study performed to characterise mutations in *ERCC8* in Chinese patients with CS-A.

## Results

### Molecular genetic identification of patients with CS-A

**Figure 1:**
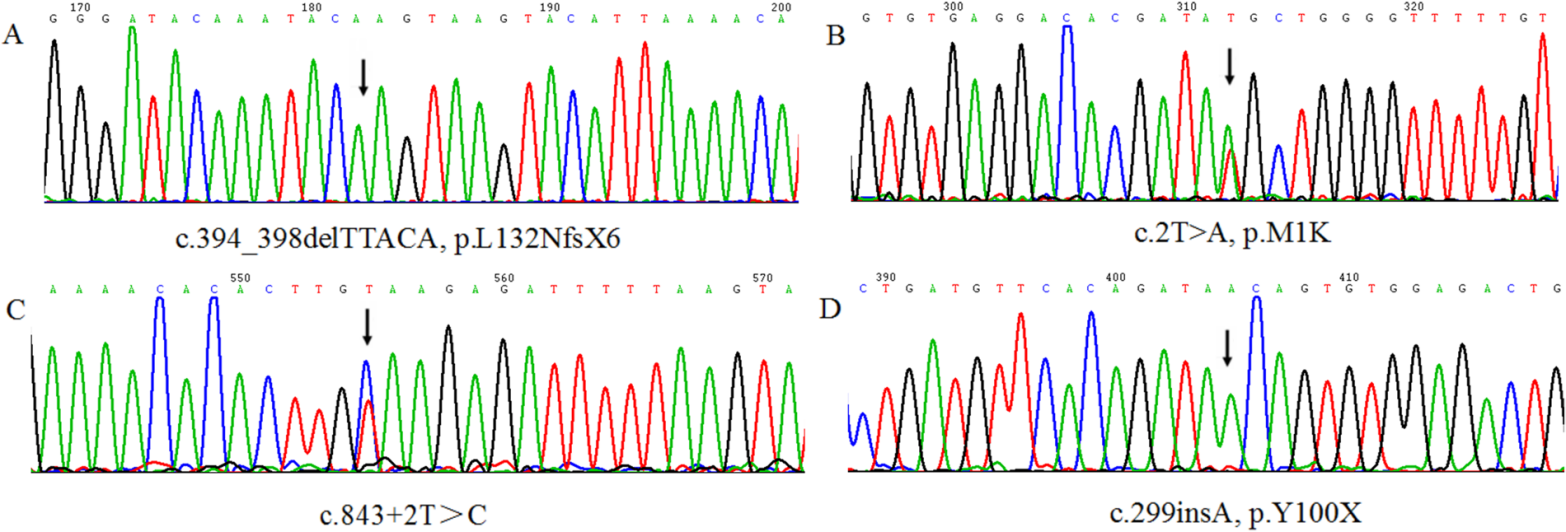
One reported mutation and three novel *ERCC8* mutations identified in a Chinese population. (**A**) The homozygous mutation c.394_398delTTACA in CS_12. (**B**) The heterozygous mutation c.2 T > A identified in CS_11. (**C**) The c.843 + 2 T > A mutation identified in CS_21, and (**D**) the mutation c.299insA, p.Y100fsX1 identified in CS_06.

Using molecular analysis, we found that 13 out of 21 patients (61.90%) in our cohort have CS-A with mutations in *ERCC8* (CS-A), 5 patients have CS-B with mutations in *ERCC6*, and 3 patients with CS lacking mutations in both genes (unpublished data). Except for CS_12, all patients with CS-A have the exon 4 rearrangement, of which six were homozygous (CS_03, CS_08, CS_14, CS_18, CS_19, and CS_20) and six were heterozygous (CS_01, CS_06, CS_07, CS_11, CS_15 and CS_21 (see Table 1). There were no PCR product with *ERCC8* exon4 primers in homozygous rearrangement mutation, such as CS_03 (see Supplementary Fig. S1A); the screening results of the pedigree of CS_03 and the other CS-A patients with primer CSA112–113 and primer CSA114-115 were showed in supplementary Fig. S1B and C. There were no PCR product with primer CSA112-113 and primer CSA114-115 of CS_12 without exon4 rearrangement mutation. In all, the exon 4 rearrangement accounts for 69.23% of all *ERCC8* alleles. In our study, we found that the c.394_398delTTACA mutation is homozygous in CS_12 and heterozygous in CS_01 (see Table 1), and accounts for 11.53% of all *ERCC8* alleles. We also discovered three novel mutations, c.299insA (p.Y100fsX1) in CS_06, c.2 T > A (p. M1L) in CS_11, and c.843 + 2 T > C in CS_21 (see Fig. 1), which were not previously reported in other populations. A single mutation was detected in CS_07 and CS_15 whose other alleles were not detected any mutation. All mutations found in our 13 patients with CS-A was presented in Table 1 and the frequencies of the mutations in *ERCC8* are presented in Table 2.

**Table 1:** Phenotypes and genotypes of all CS-A patients.

Patient ID	Mutations on genomic DNA	Protein (predicted)	Growth failure	Low birth weight	Cachexiali-podystrophy	Mental retardation (S/M)	Micro-Cephaly(C/P)	Micro-phthalmia	Vision Decrease	Hearing decrease	photo-sensitivity	Dental anomalies	Unable to Walk	protruding ears	Age at onset	Age at death or latest report
CS_01	exon 4 rearrangement^e^	p.D93LfsX26	+	−	−	S	P	+	+	+	+	−	+	+	12m	3y
	c.394_398delTTACA	p.L132NfsX6														
CA_03	exon 4 rearrangement	p.D93LfsX26	+	−	+	S	P	+	+	−	+	+	+	+	12m	7y
	exon 4 rearrangement	p.D93LfsX26														
CA_06	c.299insA	p.Y100X	+	−	+	S	P	+	+	+	+	+	+	+	8m	10y
	exon 4 rearrangement	p.D93LfsX26														
^f^CA_07	exon 4 rearrangement	p.D93LfsX26	?	?	?	?	?	?	?	?	?	?	?	?	?	?
	?^g^	?														
CA_08	exon 4 rearrangement	p.D93LfsX26	+	−	+	S	P	+	−	−	+	−	+	+	8m	6y
	exon 4 rearrangement	p.D93LfsX26														
^f^CA_11	c.2 T > A	p.M1L	?	?	?	?	?	?	?	?	?	?	?	?	?	?
	exon 4 rearrangement	p.D93LfsX26														
CA_12	c.394_398delTTACA	p.L132NfsX6	+	−	−	M	P	+	+	+	+	−	need help	+	12m	6y
	c.394_398delTTACA	p.L132NfsX6														
CA_14	exon 4 rearrangement	p.D93LfsX26	+	−	+	S	P	+	−	−	+	−	+	+	14m	6y
	exon 4 rearrangement	p.D93LfsX26														
CA_15	?^g^	?	+	−	+	M	P	+	+	+	+	−	Walk unsteady	+	4y	13y
	exon 4 rearrangement	p.D93LfsX26														
CA_18	exon 4 rearrangement	p.D93LfsX26	+	−	+	S	S	+	+	+	+	+	+	+	6m	13y
	exon 4 rearrangement	p.D93LfsX26														
CA_19	exon 4 rearrangement	p.D93LfsX26	+	−	+	S	P	+	−	−	+	+	+	+	1y	7y
	exon 4 rearrangement	p.D93LfsX26														
CA_20	exon 4 rearrangement	p.D93LfsX26	−	−	−	S	P	+	+	+	++	−	+	−	1y	7y
	exon 4 rearrangement	p.D93LfsX26														
CA_21	exon 4 rearrangement	p.D93LfsX26	+	−	+	M	P	+	+	+	+	+	Need help	+	6m	13y
	c.843+2T>C	?														

^a^Moderate (M) or severe (S).

^b^Congenital (C) or postnatal (P).

^c^Yes (+), no (−).

^d^Age at death. m, months; y, years.

^e^Exon 4 rearrangement = c.[275 + 703_399 + 347del; 399 + 348_399 + 2007inv; 399 + 2008_399 + 2558delins8] (large deletion of exon 4 and inversion in intron 4).

^f^Not unavailable clinical document.

^g^Undetected mutation in another allele.

**Table 2:** The types and frequency of *ERCC8* mutations.

The types of *ERCC8* mutation (26)	The frequency of each mutation
E4 rearrangement mutation (18)	69.23%
c.394_398delTTACA, p.L132NfsX6 (3)	11.53%
c.299insA, p.Y100X (1)	3.85%
c.2 T > A, p.M1L (1)	3.85%
c.843 + 2 T > C (1)	3.85%
undetected mutation (2)	7.69%

### The structure of the exon 4 deletion mutation

**Figure 2:**
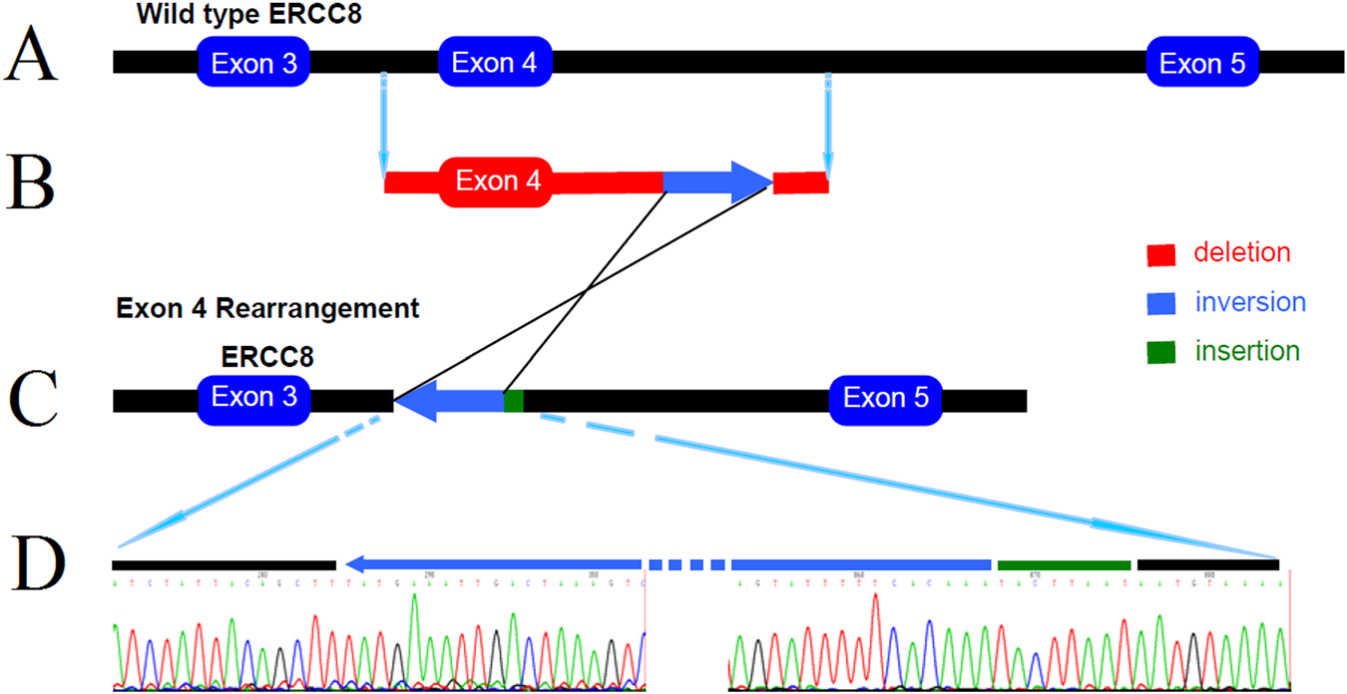
Schematic diagram of the exon 4 rearrangement mutation. (**A**) Wild-type *ERCC8*; (**B**) the affected genetic region. (**C**) The exon 4 rearrangement mutation in *ERCC8*. (**D**) Sequence analysis of the two breakpoints. Note that the red bars show two deletions consisting of a 3368-bp region in exon 4 (c.275 + 703 to c.399 + 347) and a 555-bp region in intron 4 (c.399 + 2008 to c.399 + 2558). The blue arrow indicates the 1660-bp inversion region in intron 4 (c.399 + 348 to c.399 + 2007) and the green bar shows the 8-bp insertion at the 5′ end of breakpoint.

To investigate and confirm the genetic structure of the exon 4 rearrangement, we sequenced the breakpoint regions. As shown in Fig. 2, the exon 4 rearrangement included a 3368-bp deletion of the exon 4-containing region from c.275 + 703 to c.399 + 347, a 1660-bp inversion in intron 4 from c.399 + 348 to c.399 + 2007 with a 555-bp deletion from c.399 + 2008 to c.399 + 2558, and an 8-bp insertion. The exon 4 rearrangement characterised in this study is identical to that reported in Japanese patients with CS-A^4,16^.

We screened one carrier with exon4 rearrangement from 856 control healthy individuals in Chinese population (see Supplementary Fig. S1C lane14).

### Clinical investigation

Although phenotypes and genotypes of all 13 patients with CS-A are provided in Table 1, we summarize the case findings of five patients with different mutational profiles, CS_01, CS_06, CS_12, CS_18 and CS_20.

When he first came to our lab, CS_01 was a 3-year-old boy with a one-year history of growth failure and developmental delay from a nonconsanguineous family whose brother had the same disease and died at 6 years of age. He was delivered after an uneventful pregnancy at 37 weeks of gestation with normal length and weight. His parents found moults on uncovered skin at 12 months. He had feeding difficulties and could not walk until he was 2 years old. He did not speak until 3 year-old. He had mental retardation, hearing loss, arthrogryposis, microphthalmia, and microcephaly. He had large ears, sunken eyes, and a sharp nose and chin. There was solar dermatitis on his face and his both hands appeared half-clenched because of high muscular tension in his palm and fingers. He had no dental anomalies, myopia, or cataracts (see Supplementary Fig. S2A). His father informed us that he was completely dependent on a wheelchair at 5 years of age because of severe arthrogryposis. With prenatal diagnosis he had a healthy sister at the age of 4-year-old. He was heterozygous for the exon 4 rearrangement and c.394_398delTTACA mutation.

When we met her in our lab, CS_06 was a 6-year-old girl from a nonconsanguineous family with healthy parents. She was delivered by caesarean section at 37 weeks of gestation having a normal length and weight, but was diagnosed with hypoxia at birth. At 3 months, she was found from ophthalmological examination to have sector pigment in the iris. Her parents found her left iliosacral articulation had dislocated at the age of 5 months, and at the age of 8 months, she had difficulty raising her head. Her mental and growth development was delayed compared to her contemporaries. She could not speak until 2 years old. At the age of 5 years, she could no longer walk because of arthrogryposis and suffered hearing loss at the age of 7. She had solar dermatitis, microcephaly, microphthalmia, and dental anomalies. She had sunken eyes and large ears (no photograph was provided). A physical examination at 10 years of age found she had short stature (110 cm) and a low body weight (20 kg). Neurological examination showed noticeable developmental delay and motor impairment. She was heterozygous for the exon 4 rearrangement and c.299insA mutation.

We first met CS_12 when he was 4 years old. His mother had hypertension. She conceived four times and stopped growth three times. CS_12 was delivered after an uneventful pregnancy at 37 weeks of gestation with normal length and weight. It was difficult for him to suck after birth. His parents found moults on uncovered skin at 12 months of age. He did not speak until he was 15 months old and had delayed physical development, mental retardation, microcephaly, large ears, arthrogryposis, metatarsus varus (pronounced in the right foot), microphthalmia, hypermetropia, and astigmatism. He walked with an unsteady gait and had sunken eyes and a sharp nose. At the age of 7, he had partial hearing loss. He had no dental anomalies or cataracts (see Supplementary Fig. S2B). He is homozygous for the c.394_398delTTACA mutation.

CS_18 consisted of 13-year-old male twins with short stature (both 120 cm) weighing 48 kg and 50 kg from a nonconsanguineous Chinese family. They were delivered with normal birth weights (2.25 kg and 2.5 kg). Their parents found moults on uncovered skin at 18 months of age. They could not speak until 14 months old and have severe mental retardation, and they only spoke some simple sentences, such as “I want to drink”. They could not walk unaided until 2 years old and have been fully wheelchair-dependent since 2016. They have had poor appetite since the birth. They had sunken eyes with dental anomalies (oligodontia) but did not have a prematurely aged appearance, hearing loss, myopia, microphthalmia, or cataracts (see Supplementary Fig. S2C). Both patients are homozygous for the exon 4 rearrangement mutation.

CS_20 is a 7-year-old girl from a nonconsanguineous family with healthy parents. She was delivered after an uneventful pregnancy with normal birth weights. She has had bad appetite since her birth. Her parents found severe moults on uncovered skin at 18 months of age. She had severe mental retardation, for she could not speak until 4-year-old, and only murmurs some simple words, such as “mama” or” papa”. Her ankles became deformation at the age of 2-year-old, so she could not walk unaided. Compared with other CS-A patients, her growth delay was not obvious for her stature was 120 cm and 27 kg, but her head circumference was only 45 cm. She had sunken eyes without dental anomalies. She did not have a prematurely aged appearance, hearing loss, myopia, or cataracts (see Supplementary Fig. S2D). Molecular diagnosis showed that she has homozygous exon 4 rearrangement mutation of *ERCC8* and a heterozygous mutation c.1039 C > T, p.Q347X of *ERCC2*.

### Haplotype analysis of the exon 4 rearrangement mutation indicates it is a founder mutation in Chinese population

**Figure 3:**
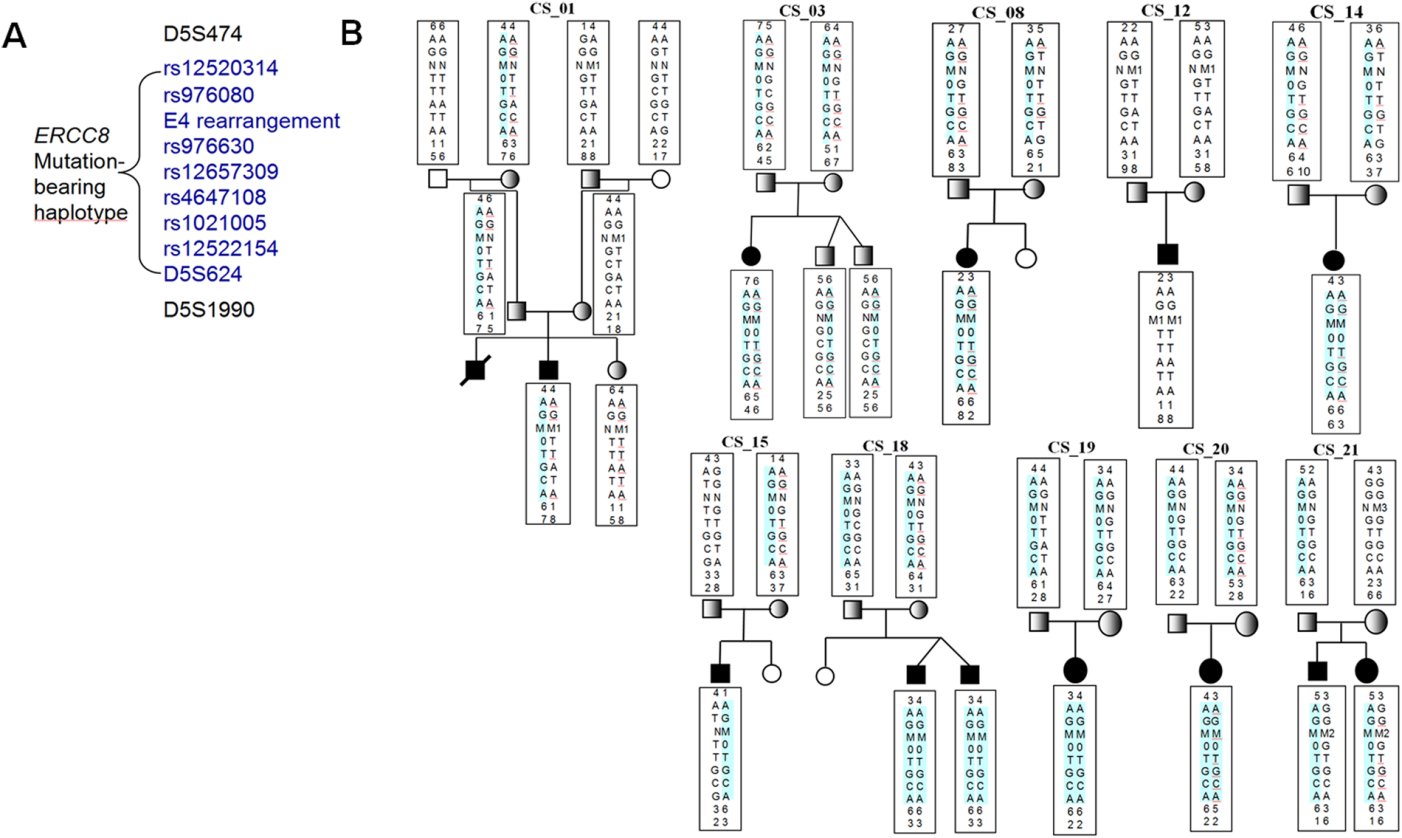
Haplotype analysis showing the exon 4 rearrangement originated from a common founder. (**A**) All polymorphic markers on chromosome 5q. (**B**) The results of haplotype analysis using nine polymorphic markers in six patients with CS-A. All exon 4 rearrangement alleles in these patients shared the same mutation-bearing haplotype (A-G-M-0-T-G-C-A), while the same c.394_398delTTACA mutation alleles were shared on the other mutation-bearing haplotype (A-G-M1-T-T-A-T-A-1-8). Except the maternal allele of CS_03 and CS_20, all the other exon4 rearrangement alleles shared the 158 bp allele of D5S624. Note that M indicates the exon 4 rearrangement; N indicates the normal (wild type) exon 4 allele; M1 represents the c.394_398delTTACA mutation; and M2 represents the c.843 + 2 T > C mutation.

To determine whether the exon 4 rearrangement mutation descended from a common founder, we selected 10 polymorphisms consisting of three STRs and seven SNPs (Fig. 3A) for haplotype construction. Using LD analysis, we found that the seven SNPs are not in the same haplotype in a Chinese population, and therefore, could be used as haplotype-tagged SNPs in this study.

We constructed haplotypes with PHASE 2.1 using seven polymorphisms (rs12520314, rs976080, rs12657309, rs4647108, rs1021005, rs12522154, and D5S624). Because rs976630 is in the deleted region of exon 4, we used “0” to substitute its genotype in the mutation-bearing haplotype. Furthermore, because rs976630 and the exon 4 deletion could not be analysed using PHASE 2.1, we substituted rs976630 with rs1021005 which are in LD for our analysis.

Genotyping results and linkage analysis showed that seven polymorphisms, consisting of all seven SNPs, are identical-by-descent with the exon 4 rearrangement mutation. We found that the alleles harboured this exon4 rearrangement shared the same haplotype with these markers (rs12520314[A]-rs976080[G]-M-rs976630[0]-rs12657309[T]-rs4647108[G]-rs1021005[C]-rs1252214[A]) (A-G-M-0-T-G-C-A) (see Fig. 3B). Of three extragenic microsatellites, 16 alleles with exon4 rearrangement shared the 158 bp allele of the marker D5S624 which located 0.11 cM from *ERCC8*, except the 156 bp of allele from maternal allele of CS_03 and CS_20. From 856 healthy individuals, we screened one carrier with exon 4 rearrangement mutation who also shared the 158 bp allele. The same 158 bp allele was present in 9 chromosomes of 220 non-carrier Chinese Han controls (110 individuals). In addition, using haplotype analysis with PHASE 2.1, we found that the mutation-associated haplotype (rs12520314[A]-rs976080[G]-rs12657309[T]-rs4647108[G]-rs1021005[C]-rs12522154[A]-D5S624[158 bp]) (A-G-T-G-C-A-158 bp) in our 12 patients and a carrier from the general population with exon4 rearrangement (n = 26 chromosomes) had a significantly higher frequency of 63.00% [standard error (SE) = 0.019331] compared to the estimated frequency of 2.27% (SE = 0.000075) found in 110 controls (n = 220 chromosomes) (*P* < 0.0001). These results strongly indicate that these alleles which bear the exon 4 rearrangement originated from a common founder.

In CS_01 and CS_12, we found the mutated allele with c.394-398delTTACA shared the same mutation-bearing haplotype (rs976080[A]-rs976080[G]-M1-rs976630[T]-rs12657309[T]-rs4647108[A]-rs1021005[T]-rs12522154[A]-D5S624[141 bp]-D5S1990[242 bp]) (A-G-M1-T-T-A-T-A-141-242) (Fig. 3B CS_01 and CS_12).

## Discussion

**Figure 4:**
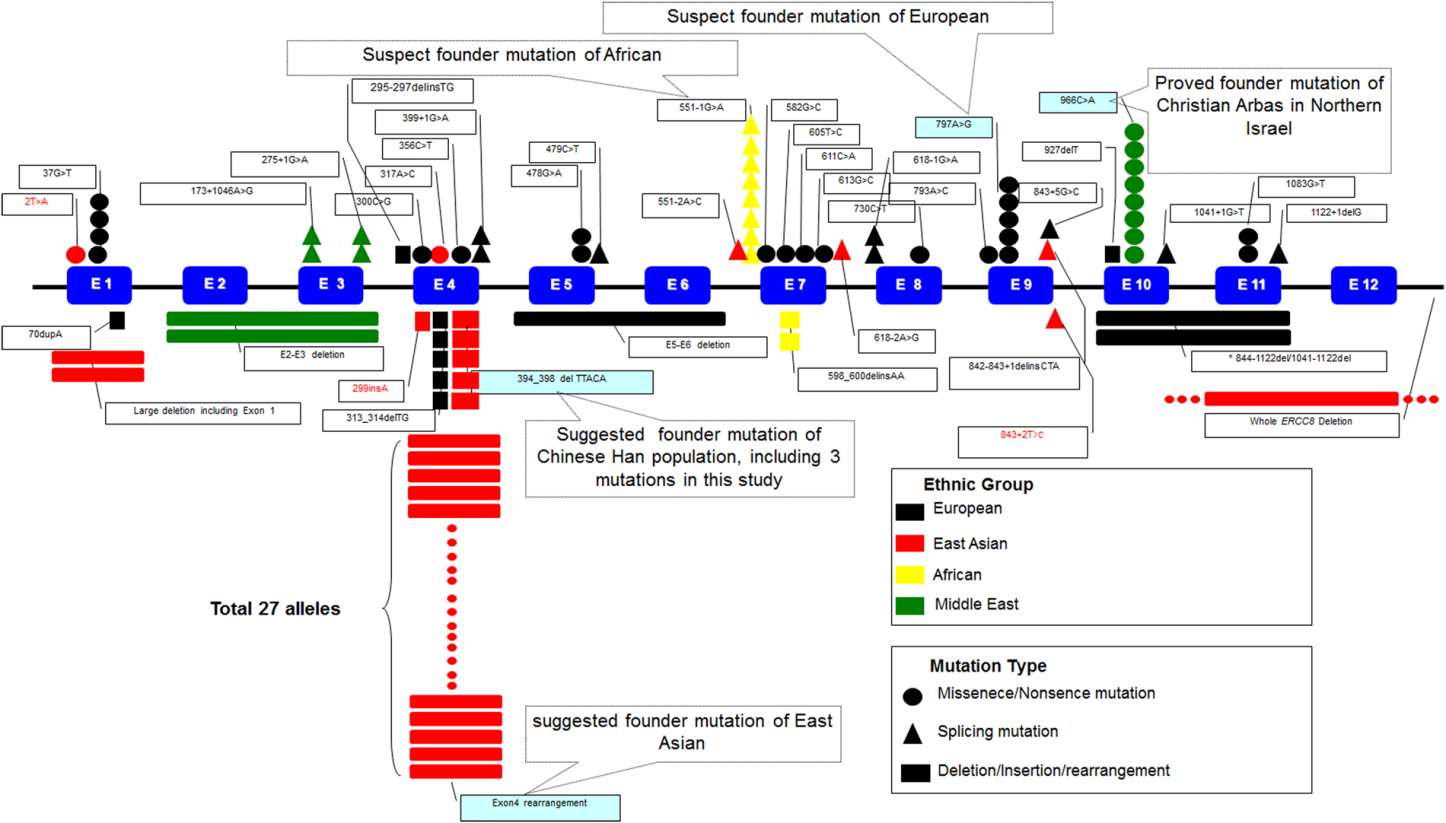
Summary of the identified mutational spectrums of *ERCC8* in different populations showing the types and numbers of each mutation in all ethnic backgrounds of *ERCC8*. Note that there are different spectrums of *ERCC8* mutations in different ethnic backgrounds. The exon 4 rearrangement mutation is a hot-spot mutation in East Asians derived from a common founder mutation (red box). The c.394_398delTTACA mutation was the second-most frequent mutation found specifically in Chinese patients with CS-A. Red word: Three novel mutations in this study.

Laugel V *et al*. summarized all the reported mutations before 2010^4^. With the development of molecular diagnosis, especially next-generation sequencing, there have been reported 12 new finding mutations reported in different ethical population since 2015, such as c.394_398delTTACA, c.561-2 A > C, the whole *ERCC8* deletion, c.295-297delinsTG, c.356 C > T, c.730 C > T, c.793 A > C, c.927delT, c. 1041 + 1 G > T, c.1122 + 1delG, c.842-843 + 1delinsCTA and c.618-2 A > G^17–22^.To date, about 40 types of mutations were found in *ERCC8* that cause CS-A in different populations, including 15 point mutations (missense and nonsense mutations), 10 splicing mutations, 3 small deletions mutations, 2 small insertion mutations, 3 small indel mutations, 5 gross deletion/duplication mutations and 1 complex rearrangement mutation, which are summarized in Fig. 4 ^4,5,15–28^.

**Figure 5:**
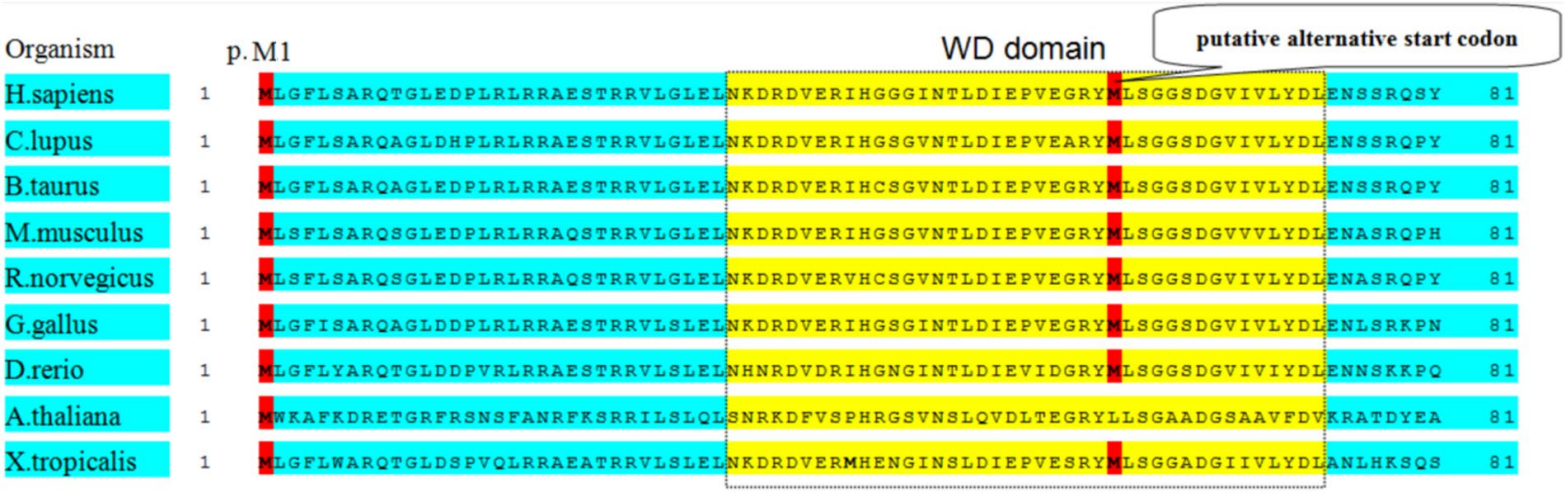
Multiple species alignment of ERCC8 orthologues shows the high evolutionary conservation of the p. M1 sequence context in vertebrates. The mutation of c.2 T > A possibly makes the translation of polypeptide chain lose the start code and result in the putative alternative start codon.

In this study, we found three novel *ERCC8* mutations which were not reported before. Our investigation of the predicted effects of these three mutations found that the c.299insA (p.Y100fsX1) mutation may confer a premature stop of the open reading frame, which could result in a truncated 100-amino-acid protein. The c.2 T > A (p.M1L) mutation possibly makes the translation of polypeptide chain lose the start code and result in the putative alternative start code (see Fig. 5), and disrupt the whole structure of CSA, which include well ordered the helix-loop-helix motif (aa 1–29) and seven WD-40 propellers (aa 30–365)^10^. The results from two prediction software programs, SIFT (Sorting Intolerant From Tolerant) and Polyphen82 (http://sift.jcvi.org) indicate that c.2 T > A is a damaging variant. The mutation of c.2 T > A is not appearing in general populations found in a SNP database including 96 healthy Han Chinese individuals. Therefore, we conclude that p.M1L is pathogenic and causal for CS-A in CS_11. The third novel mutation identified in our study is c.843 + 2 T > C, which is predicted to affect the conserved donor site (GT) of the canonical GT-AG rule, which is in the 5′ end of intron 9. It also does not appear in general populations found in a SNP database and is absent in 96 healthy Han Chinese individuals.

A single mutation (exon4 rearrangement) was detected in CS_07 and CS_15 both of whom manifested typical characteristics of CS. We analysed the haplotypes of *ERCC8* in these individuals, which showed some SNPs were heterozygous to exclude gross deletion of *ERCC8*. The result of our screening of *ERCC6/XPB/XPD* was also negative (data unpublished). So we still believe both cases are CS-A patients and need further investigation.

A previous report showed that there was a possible founder effect of the c.551-G > A mutation in the Somali population because the same mutation was only found in Somali kindred in the United Kingdom and Norway^4,26^, however the authors did not provide further proof to test the hypothesis. Another *ERCC8* mutation that results in p.Tyr322X was demonstrated by Khayat M *et al*. (2010) using haplotype analysis to be an ancient founder mutation among Christian Arabs in Northern Israel^15^.

Previous study indicated exon4 rearrangement was the major mutation in Japanese population^16^, and several Chinese CS patients carrying the mutation were also reported^18,22^. We found the exon 4 rearrangement mutation in 12 patients with CS-A, 6 of which were homozygous, and accounted for 69.23% of all alleles in our CS-A patients (18/26). So we put forward a hypothesis that the exon 4 rearrangement mutation is a founder mutation in the Chinese population. If there was a founder effect in a given population, the mutated allele should be shared on the same haplotype in the unique chromosomal background on which the mutation occurred. Haplotype analysis with linkage analysis and PHASE 2.1 suggested all these mutated alleles with exon4 rearrangement shared the mutation-bearing haplotype (A-G-M-0-T-G-C-A) and the mutation-associated haplotype (A-G-T-G-C-A-158 bp). Since this mutation has not been previously reported in other populations except for Japanese, which is also an East-Asian population, it is possible that the origin of the exon 4 rearrangement was from a common mutated founder in an East-Asian subpopulation.

Furthermore, if a mutation was from a common founder in a specific population, it should have a relatively high frequency in this population. Therefore, we screened the exon 4 rearrangement in 856 healthy individuals from a Han Chinese population, which is in Hardy-Weinberg equilibrium. We identified one person carrying this mutation, who also had the same mutation-bearing haplotype (A-G-M-0-T-G-C-A). According to this preliminary screen, the exon 4 rearrangement carrier frequency in Chinese is about 1 in 856 (0.12%).

Besides, c.394_398delTTACA was the second-most frequent mutation in our cohort, which was reported only in Chinese population^17,21^. We found that all c.394_398delTTACA alleles shared the same haplotype (A-G-M1-T-T-A-T-A-141-242) (Fig. 3B), which suggests that these alleles were also from a common founder. However, our cohort did not have enough cases to provide stronger support.

A previous study showed that patients with CS-A accounted for approximately one-third of all CS cases^4^. This is in contrast to our study in which our cohort of 21 patients with CS from a Han Chinese population included 13 patients with CS-A (61.90%) as well as 5 patients with CS-B (23.80%) and 3 patients with no discernible mutation (14.30%) in *ERCC6* or *ERCC8*. So far, no *ERCC6* founder mutation or high frequency mutation has been reported in Chinese population. The founder effects of the exon 4 rearrangement and the c.394_398delTTACA deletion may explain the higher proportion of CS-A in Chinese CS patients, although we cannot exclude the possibility that CS-B is a more serious condition with an earlier onset than CS-A and received more misdiagnosis.

A review of available clinical data showed some overlap in the clinical phenotypes between CS-A and CS-B in our cohort of CS patients. Although the focus of this study was on CS-A, our results will aid in developing a practical genetic diagnostic strategy tailored for Chinese patients with CS. Because the exon 4 rearrangement is difficult to detect by Sanger sequencing and next-generation sequencing, we recommend for Chinese and East-Asian patients with CS to initially screen for the two *ERCC8* mutations: the exon 4 rearrangement and c.394_398delTTACA.

## Conclusions

The spectrum of *ERCC8* mutations of Chinese is different from that of other populations. We found that the exon 4 rearrangement mutation and c.394_398delTTACA were the major mutations present in Han Chinese patients with CS-A, and also discovered three novel mutations in our Chinese cohort of CS-A. We propose that there was a founder effect of the exon 4 rearrangement in Chinese and East-Asian populations, which will guide development of a practical genetic diagnostic strategy for Chinese and East Asian patients with CS.

## Materials and Methods

### Ethics Statement

The study was performed in accordance with the Declaration of Helsinki and approved by the Peking University Biomedical Ethics Committee in China (approval number IRB00001052-2014028). Twenty-one unrelated patients with CS from the Northern Han Chinese population were included in the study and were anonymized as CS_01 to CS_21. Written informed consent was obtained from the patients’ parent for the publication of this clinical information. Informed consent was obtained from all subjects.

### Molecular analysis of *ERCC8*

Genomic DNA was isolated from peripheral blood using a DNA isolation kit (Aidelai, China) according to the manufacturer’s protocol. The entire coding region and flanking intron-exon boundaries of *ERCC8* underwent direct sequencing for analysis. Primers for sequencing these regions of *ERCC8* were designed by primer3.0 and all primer sequences are available upon request. The complete *ERCC8* genomic DNA sequence is NG_009289.1. The transcription isoform number is NM_000082.3. PCR reactions were performed in 25 μL reaction volumes containing 50 ng genomic DNA, 5 pmol of each primer, and 1× Taq mix (Aidelai). PCR was performed on an ABI 9800 (Perkin-Elmer Applied Biosystems, Foster City, CA, USA) using a 3-step cycle protocol consisting of an initial 5 min denaturation at 95 °C followed by 35 cycles of 95 °C for 45 s, 50 °C for 45 s, and 72 °C for 45 s, and a final extension for 10 min at 72 °C. PCR products were separated by electrophoresis on 3% agarose gels. PCR products were purified to remove primers and dNTPs prior to sequencing using an ABI Prism 3100 (Perkin-Elmer Applied Biosystems). Sequence data of PCR products were analyzed using Chromas 2.22.

To investigate the exon 4 rearrangement mutation, we used primers CSA112-113 and CSA114-115 to screen all patients and primers CSA_60-62 to amplify and sequence the mutant alleles^16^.

### Linkage and haplotype analysis

To study haplotype segregation in our patients with CS-A and Han Chinese controls, we selected 10 polymorphic sites consisting of three extragenic microsatellites, the telomeric marker D5S474 and two centromeric markers, D5S624 and D5S1990, located 0.2 cM, 0.11 cM, and 0.6 cM from *ERCC8*, respectively^15,28^ as well as seven intragenic Haplotype-tagged SNPs (rs12520314, rs976080, rs976630, rs12657309, rs4647108, rs1021005, and rs12522154) identified from HapMap (https://www.ncbi.nlm.nih.gov/variation/tools/1000genomes/). Linkage disequilibrium (LD) analysis was performed using Haploview 4.0.

Standard PCR amplification of microsatellites was performed using fluorescently labelled primers to analyse PCR products on an ABI Prism 3100 (Perkin-Elmer Applied Biosystems), which was also use to sequence the PCR products of our intragenic SNPs. The sequences of the primers used for SNP and microsatellite sequencing are available upon request. Haplotypes analysis was performed using PHASE version 2.1 (http://stephenslab.uchicago.edu/phase/download.html).

All methods were carried out in accordance with relevant guidelines and regulations and all experimental protocols were approved by the Peking University Biomedical Ethics Committee in China.

### Ethics approval and consent to participate

The study was approved by the Peking University Biomedical Ethics Committee in China (approval number IRB00001052-2014028).

### Consent to publish

Written informed consent was obtained from the patient’s parents for publication of this paper. A copy of the written consent is available for review by the Editor of this journal.

### Availability of data and materials

All the data can be available upon requested.

## Electronic supplementary material

Supplementary Information
