# Supplementary material for: Molecular spectrum of excision repair cross-complementation group 8 gene defects in Chinese patients with Cockayne syndrome type A
Source: Sci Rep. 2017 Oct 20;7:13686. doi: 10.1038/s41598-017-14034-3 (PMC5651726; doi:10.1038/s41598-017-14034-3)
Supplement: Supplementary file 1 — Supplementary Information [file 41598_2017_14034_MOESM1_ESM.pdf]

Molecular spectrum of excision repair cross-complementation group 8 gene defects in Chinese patients with Cockayne syndrome type A

Short title: *ERCC8* mutations in Chinese patients with CS-A

Xiaozhu Wang†[1,2], Yu Huang†[2], Ming Yan[2], Jiu Wei Li[3], Changhong Ding[3], Hong Jin[3], Fang Fang[3], Yanling Yang[4], Baiyan Wu[2], Dafang Chen*[1]

[1]Department of Epidemiology and Biostatistics, School of Public Health, Peking University Health Science Center, Beijing, China, 100191

[2]Department of Medical genetics, School of Basic Medical Sciences, Peking University Health Science Center, Beijing, China, 100191

[3]Department of Neurology, Beijing Children's Hospital, Capital Medical University, Beijing, China, 100045

[4]Departments of Paediatrics, Peking University First Hospital, Beijing, China**,** 100034

†These authors contributed equally to this work

*Corresponding author: Dafang Chen, 38 Xue Yuan Road, Haidian District, Beijing, 100191, China Tel: 86-10-8280-2644; Email: dafangchen@bjmu.edu.cn

Fig S1

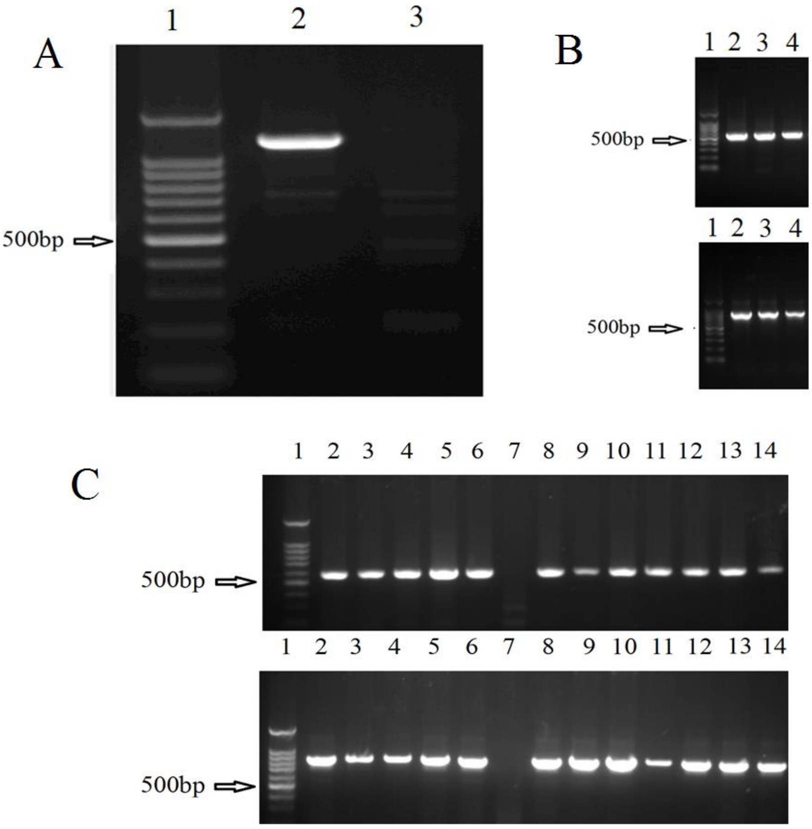

Fig S1 A-C: A showed the PCR products of *ERCC8* E4, the lane 1 showed the marker(100bp), lane 2 showed the PCR product of exon 4 of normal control; lane 3 showed homozygous deletion exon 4 of CS_03. B showed the PCR products with primer CSA112-113 and CSA114-115 of the pedigree of CS_03, the lane 1 showed the marker(100bp), lane2-4 showed the products of CS_03, her father and her mother. C showed the PCR products with primers CSA112 -113 and 114-115. Lane 1 showed the marker (100bp), lane 2-14 showed the products of CS_01, CS_06, CS_07, CS_08, CS_11, CS_12, CS_14, CS_15, CS_18, CS_19, CS_20, CS_21and the carrier from normal Chinese individuals. There were no PCR product with primer CSA112-113 and 114-115 of CS_12 who was homozygous mutation of c.394_398delTTACA.

Fig S2

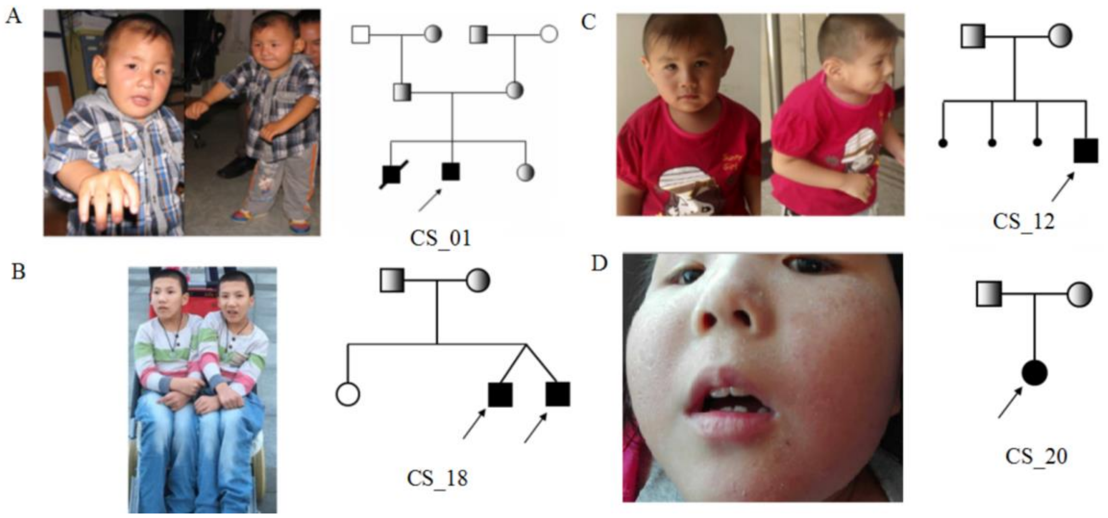

Fig S2A-D: A: Phenotypes and pedigrees of CS_01; B: Phenotypes of CS_12; C: Phenotypes and pedigrees of CS_18; D: Phenotypes and pedigrees of CS_21, all of which had different *ERCC8* mutation profiles.
